# Complete plastome sequence of *Lonicera gynochlamydea* Hemsl. (Caprifoliaceae)

**DOI:** 10.1080/23802359.2022.2101396

**Published:** 2022-07-28

**Authors:** Rong-Jia Mo, Hong-Xin Wang, Da-Juan Chen, Zhi-Xin Zhu, Wang Qiao, Hua-Feng Wang

**Affiliations:** a Sanya Nanfan Research Institute of Hainan University, Sanya, China; b Zhai Mingguo Academician Work Station, Sanya University, Sanya, China; c Hainan Grand Modern Agriculture Development Co. Ltd., Qionghai, China

**Keywords:** Caprifoliaceae, genome structure, *Lonicera gynochlamydea*, plastome

## Abstract

We report and characterize the complete plastome of *Lonicera gynochlamydea* Hemsl*. L. gynochlamydea* is a shrub, belonging to the family Caprifoliaceae. Our results show that the length of the complete plastome is 154,643 bp, including 131 genes consisting of 84 protein-coding genes, 39 tRNA genes, and eight rRNA genes. The plastome exhibits the typical quadripartite structure and gene content of angiosperms, composed of two inverted repeats (IRs) regions of 23,846 bp, a large single-copy (LSC) region of 88,298 bp, and a small single-copy (SSC) region of 18,653 bp. The total G/C content in *L. gynochlamydea* plastome is 38.4%. The complete plastome sequence of *L. gynochlamydea* will make contributions to the conservation genetics of this species as well as to phylogenetic studies in Caprifoliaceae.

*Lonicera gynochlamydea* Hemsl. 1888 is a deciduous shrub belonging to the family Caprifoliaceae. The species is distributed in southern Shaanxi and Gansu, southern Anhui (Guichi), western Hubei, northwestern Hunan (Sangzhi), northern Sichuan (Pingwu), and northeastern and western Guizhou (Bijie). Plants grow in thickets or forests on slopes and valleys at an altitude of 1200–1900 (–3000) m above sea level. *L. gynochlamydea* has high ornamental value with a distinct aroma (Zheng et al. 2009). At the same time, *L. gynochlamydea* also has certain medicinal value, commonly used in folk herbs in China (Yan et al. 2013) primarily due to its heat-clearing, detoxification, and efficacy in treating dysentery. Therefore, we report the complete plastome of *L. gynochlamydea* in this study, which is expected to improve the quality of relevant collections, medical applications and phylogenetic investigations of Caprifoliaceae.

In this study, *L. gynochlamydea* was sampled from the city of Jinzhai in An'hui province (115°20′E, 31°10′N). The voucher specimen (voucher code: H F Wang, L316, HUTB) and its DNA were deposited in the Herbarium of the Institute of Herbarium of China National GenBank (code of herbarium: HUTB). The experiment was carried out as reported in Zhu et al. (2018). Clean sequence data were assembled with GetOrganelle v1.7.5.0 (Jin et al. 2020). We used Geneious Prime v2021.2.2 (Biomatters Ltd., Auckland, New Zealand) to annotate the assembled plastome based on the reference of *L. ruprechtiana* Regel (NC_056986), and this annotation was corrected with DOGMA (Wyman et al. 2004).

Our results show that the plastome of *L. gynochlamydea* bears the typical quadripartite structure of angiosperms and is a total of 154,643 bp in length. The plastome consists of two inverted repeats (IRs) of 23,846 bp each, a large single-copy (LSC) region of 88,298 bp, and a small single-copy (SSC) region of 18,653 bp. The plastome contains 131 genes, including 84 protein-coding genes (five of which are repeated in IR), 39 tRNA genes (eight of which are repeated in IR), and eight rRNA genes (5S rRNA, 4.5S rRNA, 23S rRNA, and 16S rRNA) (four of them are repeated in IR). The total G/C content in *L. gynochlamydea* plastome is 38.4%. The corresponding G/C values of LSC, SSC, and IR regions are 36.9%, 33.2%, and 43.3%, respectively.

We used RAxML (Stamatakis 2006) with 1000 bootstraps under the GTRGAMMAI substitution model to reconstruct a maximum-likelihood (ML) phylogeny of 12 published complete plastomes of Caprifoliaceae, using *Sambucus adnata* NC_051521.1 and *Viburnum farreri* NC_056112.1 as outgroups. By inferring phylogenetic relationships based on the existing data and related taxa, we find that *L. gynochlamydea* is sister with the clade of *L. maackii* Maxim. (*L. insularis* Gary, *L. ruprechtiana* Regel) in this study (Figure 1). Most nodes in the plastome ML tree were highly supported. Comparison of the *L. gynochlamydea* plastome to previously published data shows a high level of gene synteny with all publicly available *Lonicera* species. This finding was similar to those of Hu et al. (2018), Kang et al. (2018), and Wang et al. (2020). The resource utilization and conservation of the species can be better supported based on the complete plastome sequence of *L. gynochlamydea*, as well as the taxonomic and evolutionary studies of *Lonicera* can be explored more thoroughly.

**Figure 1.**
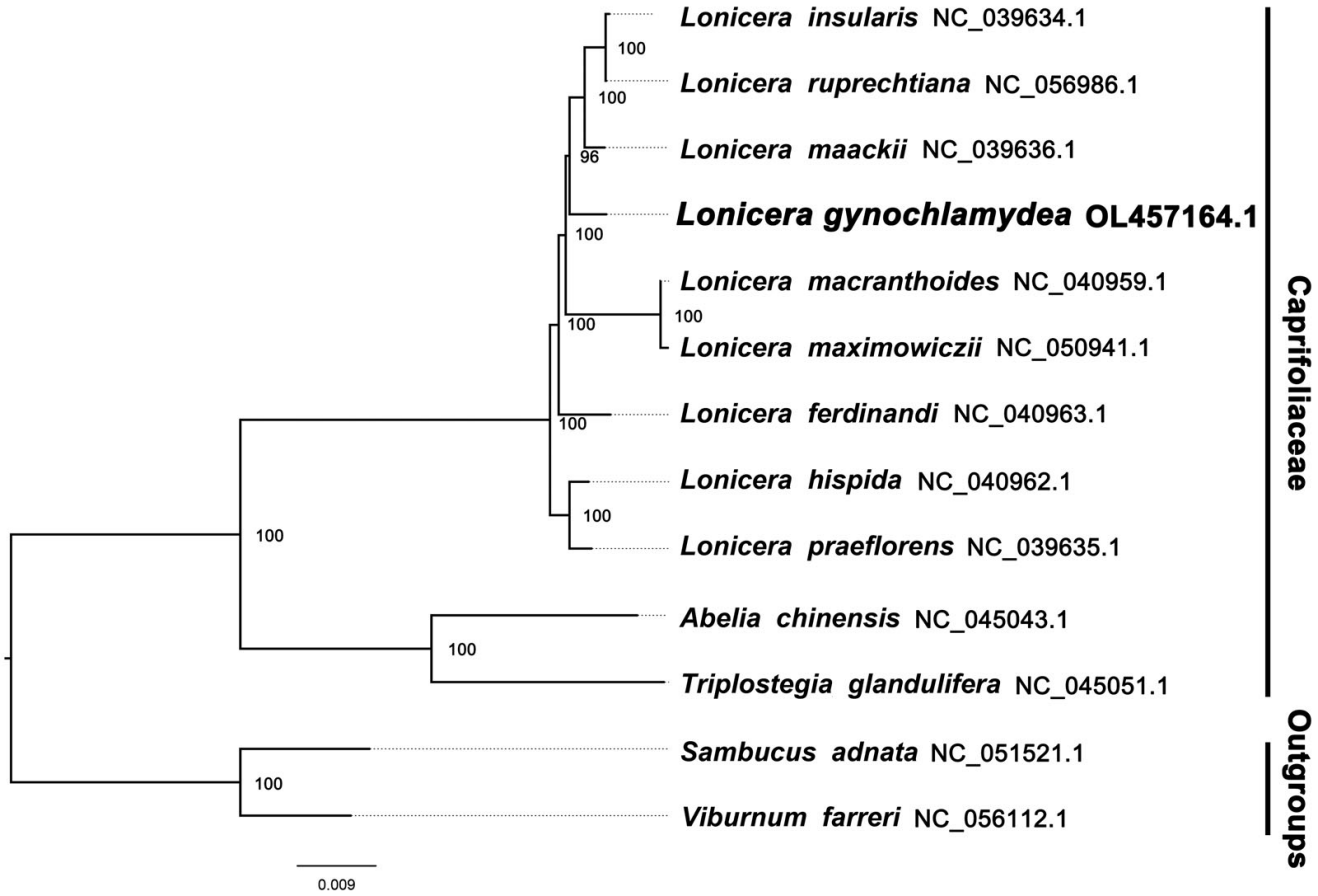
The ML phylogeny recovered from 13 complete plastome sequences using RAxML.

## Data Availability

The genome sequence data supporting the results of this study were deposited in GenBank of NCBI (https://www.ncbi.nlm.nih.gov/) with accession number OL457164.1. The associated BioProject, SRA, and Bio-Sample numbers are PRJNA748537, SRR15533081, and SAMN20703094, respectively.
